# Mediastinal effusion due to pericardiocentesis with cardiac tamponade: a case report

**DOI:** 10.1186/s12871-021-01385-8

**Published:** 2021-06-16

**Authors:** Qian Zhang, Difen Wang, Ying Liu

**Affiliations:** Department of Intensive Care Unit (ICU), The Affiliated Hospital of Guizhou Medical University, 550004 Guiyang, Guizhou P.R. China

**Keywords:** Mediastinal effusion, Pericardiocentesis, Cardiac tamponade, Pericardial effusion, Inferior vena cava filter.

## Abstract

**Background:**

Pericardiocentesis is an effective treatment for cardiac tamponade, but there are risks, including haemorrhagic events, cardiac perforation, pneumothorax, arrhythmia, acute pulmonary oedema and so on. Mediastinal effusion caused by puncture is rarely reported.

**Case presentation:**

A 47-year-old man who had a history of right leg deep vein thrombosis and pulmonary artery embolism with implantation of an inferior vena cava filter presented for inferior vena cava filter removal. Within 30 min after the procedure, he developed chest pain, nausea, vomiting and presyncope with shock. Echocardiography confirmed massive pericardial effusion with evidence of cardiac tamponade. Emergency pericardiocentesis was performed. Confusingly, only 3 mL of bloody pericardial effusion was drained in total, and subsequently, the patient’s symptoms rapidly improved with stable haemodynamics. Repeat echocardiography showed that the pericardial effusion had disappeared. Urgent computed tomography pulmonary angiography demonstrated localized effusion, which was not seen the previous computed tomography results and was noted around the left ventricle in the mediastinal apace. No intervention was performed, given that there was no bleeding tendency or further adverse events related to the mediastinal effusion. The patient was subsequently discharged in a stable condition a few days later, and outpatient follow-up was advised.

**Conclusions:**

Mediastinal effusion is a rare complication of pericardiocentesis. In the case described herein, the most likely cause was pericardial effusion extravasated into the mediastinum through the needle insertion site in the puncture process due to large pressure variations in the intrapericardial space with tamponade, differing from cases of over-anticoagulation reported in the previous literature. Just as our case demonstrates that conservative treatment of an hemodynamic insignificant mediastinal effusion may be appropriate. Echocardiography is useful and effective to minimize complication rates.

**Supplementary Information:**

The online version contains supplementary material available at 10.1186/s12871-021-01385-8.

## Background

Cardiac tamponade is a critical condition that requires immediate intervention. [1] Pericardiocentesis is an effective but risky management approach, sometimes leading to serious procedure-related complications, such as cardiac puncture, pneumothorax, arrhythmia and acute pulmonary oedema. [2] However, among the reported complications, mediastinal effusion is very rare. We present a case of a patient who experienced mediastinal effusion due to pericardiocentesis with cardiac tamponade following inferior vena cava filter removal.

## Case presentation

**Fig. 1:**
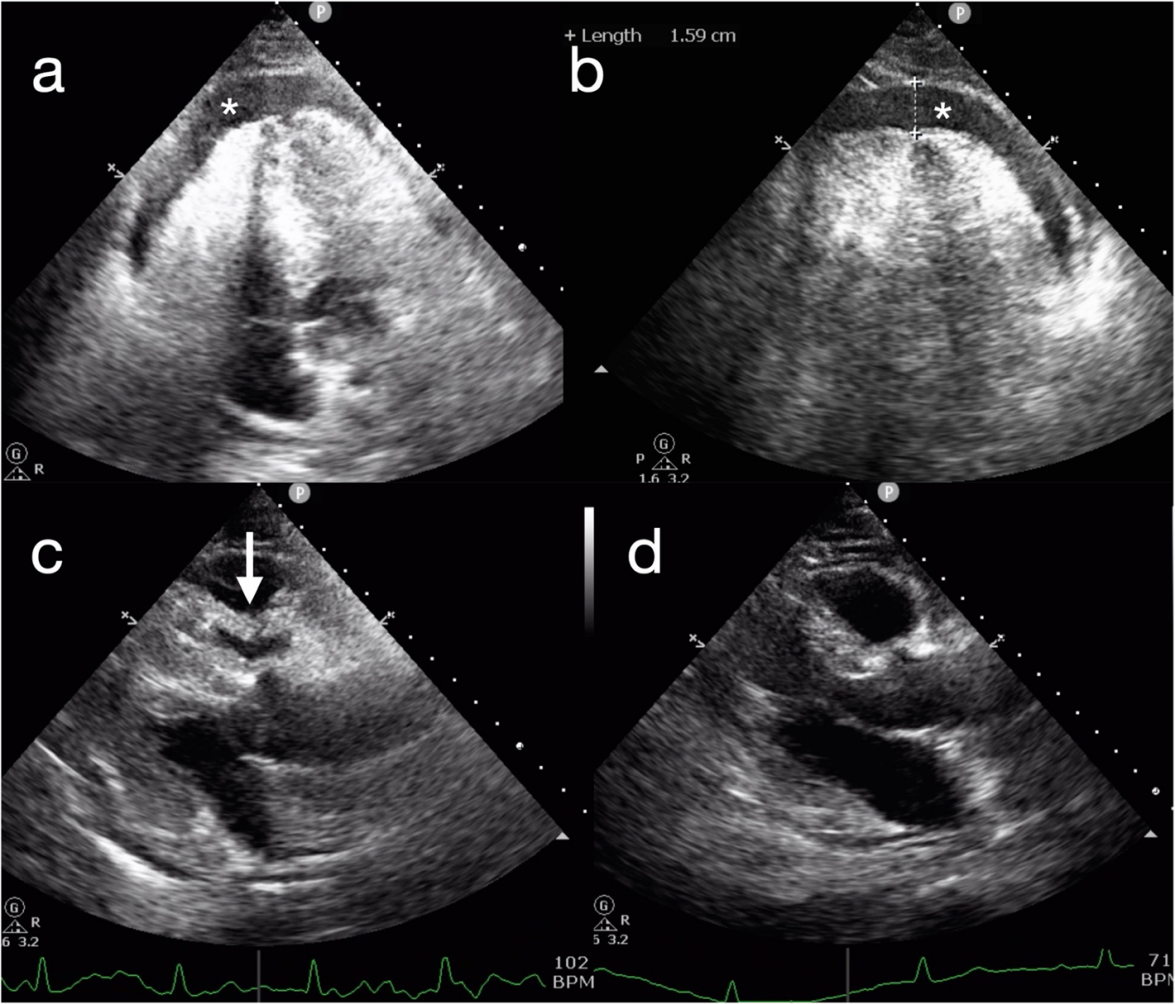
Echocardiography with a four-chamber view and a parasternal long axis. Circumferential pericardial effusion (*) (Panel **a**) was 1.59 cm in the largest dimension (Panel **b**). Sign of cardiac tamponade (Panel **c**): diastolic collapse of the right ventricle with pericardial effusion (white arrow). The pericardial effusion disappeared after the pericardiocentesis (Panel **d**)

**Fig. 2:**
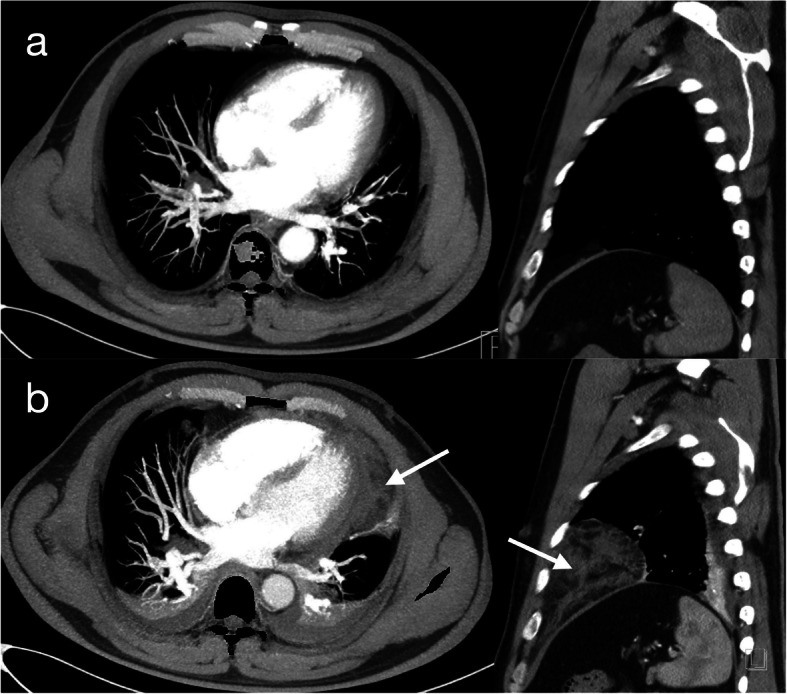
Computed tomography pulmonary angiography No mediastinal effusion occurred before the pericardiocentesis (Panel **a**). The white arrow shows the mediastinal effusion after the pericardiocentesis (Panel **b**)

A 47-year-old man presented for inferior vena cava (IVC) filter removal, which was initially placed roughly one month prior to this hospitalization. He had a 10 pack-year history of smoking and was diagnosed with thromboangiitis obliterans for more than six months. At that time, the patient had right leg deep vein thrombosis (DVT) and bilateral segmental pulmonary embolism (PE). A nonpermanent IVC filter was placed to prevent recurrence of PE from DVT, and the DVT was treated with mechanical thrombectomy. Subsequently, the patient received long-term anticoagulation with rivaroxaban. In the present hospitalization, computed tomography pulmonary angiography (CTPA) showed that the bilateral segmental pulmonary thrombus had almost disappeared, and ultrasonography showed partial thrombus dissolution and patency of blood flow in the vein of the right lower limb. Based on the above results, it was not necessary to retain the filter. Then, interventional radiology-guided IVC filter removal was performed in the operating room. Under X-ray fluoroscopy, a snare was inserted into the right internal jugular vein, and the IVC filter was withdrawn from its hook. The procedure was uneventful. Within 30 min after the procedure, the patient developed chest pain, nausea, vomiting and presyncope. Physical examination showed a blood pressure of 70/34 mmHg, a pulse of 108 beats per minute, and a respiratory rate of 23 breaths per minute. Arterial blood gas showed a pH of 7.31, PaCO_2_ of 36 mmHg, PaO_2_ of 68 mmHg, HCO_3_ of 18.1 mmol/L, lactate 5.8 mmol/L and oxygen saturation of 95 %. Bedside echocardiography confirmed circumferential pericardial effusion, 1.59 cm in the largest dimension, with evidence of cardiac tamponade (Fig. 1, videos in supplementary files 1 and 2). The ideal puncture site, as defined by echocardiography, was para-apical. Pericardiocentesis was immediately performed with an 18G (1.3 × 1.06 × 65) mm needle after echocardiography localization. Confusingly, only 3 mL of bloody pericardial effusion was removed in total, and subsequently, the patient’s symptoms significantly improved except for mild subxiphoid pain. His vital signs improved approximately 3 min later, with a blood pressure of 125/95 mmHg, a pulse of 70 beats per minute, a respiratory rate of 20 breaths per minute, and an oxygen saturation of 99 %. Clotting tests indicated an INR of 1.15, an activated partial thromboplastin time of 34.7 s, and a prothrombin time of 14.5 s. Repeat echocardiography revealed that the pericardial effusion had disappeared (Fig. 1d, video in supplementary file 3). To clear up the confusion, an urgent CTPA was performed approximately 3 h after the procedure. The images showed mediastinal effusion, which had not been seen on the previous CTPA (Fig. 2a), around the left ventricle in the anterior mediastinum (Fig. 2b). Considering that the patient was asymptomatic and organ failure secondary to mediastinal effusion were not present, no intervention was suggested. He remained in the hospital for three days after the procedure. He was subsequently discharged in a stable condition, and outpatient follow-up was advised.

## Discussion and conclusions

The indication for IVC filter implantation is still debatable. Societal guidelines vary in the indications. [3–5] The most widely accepted one is the prevention of PE in a patient with DVT who cannot receive anticoagulation. Other accepted indications for IVC filter implantation include a complication of anticoagulation, thrombus progression despite adequate anticoagulation, high-risk or massive PE, and free-floating thrombosis or large acute thrombosis in iliac, femoral vein or inferior vena cava. In our case, the patient had large acute right leg DVT and bilateral segmental PE. A IVC-filter was indicated to intercept thrombus that had broken free from lower limb DVT during mechanical thrombectomy and prevent its migration to the lungs. In current practice, the retrievable filters are placed much more commonly than the permanent filters. The retrievable IVC filter should be removed once placement indications are no longer present.

IVC filter removal can lead to various complications, including IVC perforation, air embolism, pneumothorax or filter migration. However, as this case and a literature review illustrates, cardiac tamponade is a rare but life-threatening complication of IVC filter removal. The likely mechanism is myocardial rupture, as presented in a previous case report. [6] In our case, although there was no definitive evidence of myocardial rupture, we thought that this might have occurred due to the possible puncture of the inferior vena cava surrounded with pericardium or right atrium by the guidewire or filter during the manipulation of the vasculature.

Echocardiography should be obtained immediately if cardiac tamponade is suspected. [7] A common sign of cardiac tamponade with significant haemodynamic compromise is collapse of the right atrium and the right ventricle. This occurs during the diastolic phase, when the intrachamber pressures are lower than the intrapericardial pressures. [8, 9] Acute tamponade generally occurs within minutes and requires urgent pericardiocentesis. Pericardiocentesis has potential risks, with major procedural complications including haemorrhagic events, cardiac perforation, pneumothorax, and arrhythmia. [10] In our case, at least 100 ml of fluid accumulated in the pericardial cavity according to the echocardiographic images; however, only 3 ml was aspirated, and the symptoms and vital signs of cardiac tamponade improved rapidly after a few minutes. These findings suggest that the tamponade had been relieved, as shown by repeat echocardiography. “Where did the effusion go?” was the question about which we were confused. Approximately 3 h after the procedure, a repeat CT scan revealed fluid accumulation in the mediastinal space.

However, mediastinal effusion due to this procedure is very uncommon. We conducted a thorough search of the literature published to date with the search terms ‘pericardiocentesis’ and ‘Mediastinal effusion or Mediastinal hematoma’ on PubMed. There are no similar published cases. In a series of 161 patients with cardiac tamponade, Maggiolini et al. [2] reported one patient whose clotting tests indicated overanticoagulation with warfarin who developed this complication within 2 days after pericardiocentesis, requiring thoracic surgery. However, this is different from our case, where our patient had no bleeding tendencies. In our case, the most likely cause was that the pericardial effusion moved into the mediastinum through the needle insertion site due to the changes in the intrapericardial pressure in response to the tamponade. When the intrapericardial pressure increases excessively due to cardiac tamponade, a bloody effusion may rapidly flow from the pericardium to the surrounding low-pressure mediastinum during the pericardiocentesis process, which led to the accumulation of an effusion in the mediastinal space.

Pericardial tamponade is an uncommon complication of IVC filter removal and is an absolute indication for pericardiocentesis. It is rare, but pericardial effusion may extravasate into the mediastinum during the procedure due to large pressure variations in the intrapericardial space, increasing the risk of cardiac perforation. Just as our case demonstrates that conservative treatment of an hemodynamic insignificant mediastinal fluid collection may be appropriate. Echocardiography is useful before, during and after the procedure to reduce the incidence of procedural complications. [2].

## Supplementary Information

**Additional file 1:** Apical 4-chamber dynamic imaging to assess the pericardial effusion.

**Additional file 2:** Parasternal long axis dynamic imaging of the cardiac tamponade.

**Additional file 3:** Parasternal long axis dynamic imaging after the cardiac tamponade was resolved.

## Data Availability

Not applicable.

## Abbreviations

IVC: Inferior vena cava
DVT: Deep vein thrombosis
PE: Pulmonary embolism
CTPA: Computed tomography pulmonary angiography
Fig: Figure

## References

[1] Adler Y, Charron P, Imazio M, et al. 2015 ESC Guidelines for the diagnosis and management of pericardial diseases: The Task Force for the Diagnosis and Management of Pericardial Diseases of the European Society of Cardiology (ESC)Endorsed by: The European Association for Cardio-Thoracic Surgery (EACTS).[J].Eur Heart J.2015,36(42):2921–2964. 10.1093/eurheartj/ehv318 PMC7539677 26320112

[2] Maggiolini S, Gentile G, Farina A, et al. Safety, Efficacy, and Complications of Pericardiocentesis by Real-Time Echo-Monitored Procedure.[J].Am J Cardiol.:1369–74. 10.1016/j.amjcard.2016.01.043 26956635

[3] Kearon C, Akl EA, Comerota AJ, et al. Antithrombotic therapy for VTE disease: Antithrombotic Therapy and Prevention of Thrombosis, 9th ed: American College of Chest Physicians Evidence-Based Clinical Practice Guidelines.[J].Chest.2012,141(2 Suppl):e419S-e496S. 10.1378/chest.11-2301 PMC3278049 22315268

[4] Kearon C, Akl EA, Ornelas J (2016). Antithrombotic Therapy for VTE Disease: CHEST Guideline and Expert Panel. Report[J]Chest.

[5] Caplin DM, Nikolic B, Kalva SP (2011). Quality improvement guidelines for the performance of inferior vena cava filter placement for the prevention of pulmonary embolism.[. J]J Vasc Interv Radiol.

[6] Levine E, Pasha K, Song J (2019). Cardiac tamponade from a fractured inferior vena cava filter.[. J]Eur Heart J Cardiovasc Imaging.

[7] Klein AL, Abbara S, Agler DA (2013). American Society of Echocardiography clinical recommendations for multimodality cardiovascular imaging of patients with pericardial disease: endorsed by the Society for Cardiovascular Magnetic Resonance and Society of Cardiovascular Computed Tomography.[J]. J Am Soc Echocardiogr.

[8] Singh S, Wann LS, Klopfenstein HS (1986). Usefulness of right ventricular diastolic collapse in diagnosing cardiac tamponade and comparison to pulsus paradoxus.[J]. Am J Cardiol.

[9] Gillam LD, Guyer DE, Gibson TC (1983). Hydrodynamic compression of the right atrium: a new echocardiographic sign of cardiac tamponade. [J]Circulation.

[10] Akyuz S, Zengin A, Arugaslan E, et al. Echo-guided pericardiocentesis in patients with clinically significant pericardial effusion. Outcomes over a 10-year period.[J].Herz.2015,40Suppl 2:153–9. 10.1007/s00059-014-4187-x 25491665

